# Aspects on the Physiological and Biochemical Foundations of Neurocritical Care

**DOI:** 10.3389/fneur.2017.00274

**Published:** 2017-06-19

**Authors:** Carl-Henrik Nordström, Lars-Owe Koskinen, Magnus Olivecrona

**Affiliations:** ^1^Department of Neurosurgery, Odense University Hospital, Odense, Denmark; ^2^Department of Clinical Neuroscience, Division of Neurosurgery, Umeå University, Umeå, Sweden; ^3^Faculty of Health and Medicine, Department of Anesthesia and Intensive Care, Section for Neurosurgery Örebro University Hospital, Örebro University, Örebro, Sweden; ^4^Department for Medical Sciences, Örebro University, Örebro, Sweden

**Keywords:** neurocritical care, intracranial pressure, cerebral blood flow, cerebral energy metabolism, microdialysis

## Abstract

Neurocritical care (NCC) is a branch of intensive care medicine characterized by specific physiological and biochemical monitoring techniques necessary for identifying cerebral adverse events and for evaluating specific therapies. Information is primarily obtained from physiological variables related to intracranial pressure (ICP) and cerebral blood flow (CBF) and from physiological and biochemical variables related to cerebral energy metabolism. Non-surgical therapies developed for treating increased ICP are based on knowledge regarding transport of water across the intact and injured blood–brain barrier (BBB) and the regulation of CBF. Brain volume is strictly controlled as the BBB permeability to crystalloids is very low restricting net transport of water across the capillary wall. Cerebral pressure autoregulation prevents changes in intracranial blood volume and intracapillary hydrostatic pressure at variations in arterial blood pressure. Information regarding cerebral oxidative metabolism is obtained from measurements of brain tissue oxygen tension (P_bt_O_2_) and biochemical data obtained from intracerebral microdialysis. As interstitial lactate/pyruvate (LP) ratio instantaneously reflects shifts in intracellular cytoplasmatic redox state, it is an important indicator of compromised cerebral oxidative metabolism. The combined information obtained from P_bt_O_2_, LP ratio, and the pattern of biochemical variables reveals whether impaired oxidative metabolism is due to insufficient perfusion (ischemia) or mitochondrial dysfunction. Intracerebral microdialysis and P_bt_O_2_ give information from a very small volume of tissue. Accordingly, clinical interpretation of the data must be based on information of the probe location in relation to focal brain damage. Attempts to evaluate global cerebral energy state from microdialysis of intraventricular fluid and from the LP ratio of the draining venous blood have recently been presented. To be of clinical relevance, the information from all monitoring techniques should be presented bedside online. Accordingly, in the future, the chemical variables obtained from microdialysis will probably be analyzed by biochemical sensors.

## Introduction

Critical—or intensive—care medicine is defined as a branch of medicine concerned with the diagnosis and treatment of life-threatening conditions requiring invasive monitoring and advanced pharmacological or technical organ support. After a serious polio epidemic with many deaths due to insufficient respiration, the first critical care unit in the world opened at Kommunehospitalet in Copenhagen in December 1953 (1). Since then, critical care medicine has gradually developed and holds today a central position within medicine (2). Neurocritical care (NCC) emerged as a separate branch in the 1980s. It was introduced to improve outcome in serious diseases of the nervous system, e.g., stroke, cerebral trauma, brain swelling, seizures, and central nervous infections. In addition to the components included in general critical care, NCC is especially focused on problems related to intracranial pressure (ICP), cerebral blood flow (CBF), and cerebral energy metabolism (3). During NCC, information regarding these physiological and biochemical variables may be obtained, displayed bedside and included in the clinical decision making. In this review, we present some of the most widely used monitoring techniques and discuss how they may be used and interpreted to evaluate and optimize therapy.

## ICP Monitoring and Volume Regulation of the Brain

### ICP Monitoring—Principles and Techniques

In the 1950s, Nils Lundberg developed the first clinical technique for continuous monitoring of ICP (4). He used a simple technique where a catheter was inserted into the cerebrospinal fluid (CSF) of the lateral ventricle through a frontal burr hole. The ventricular cannula was then *via* a strain gage pressure transducer connected to a potentiometer recorder, and the ICP was displayed bedside online. The technique has remained the “gold standard” for ICP monitoring (5) due to several advantages: as calibration is possible, the data obtained are accordingly always correct, and CSF may be drained to decrease ICP. The technique is not without risk but serious complications like hemorrhages and intraventricular infection can be kept at a low and acceptable level (5). However, it can be technically difficult to insert the ventricular catheter in patients with compressed or dislocated ventricles.

Techniques utilizing intra-parenchymal ICP recording circumvent some of these problems. Several technical solutions are available utilizing fiberoptic tips or micro strain gages. The fiberoptic sensor utilizes the path length change induced by pressure applied to a diaphragm. The miniaturized strain gage sensors have a foil responding with a change in resistance as stress is supplied and the strain gage element is connected to a half or complete Wheatstone bridge circuit. This technique is common for many pressure transducers used in medicine. A principle difference from the intraventricular technique is the inability to recalibrate the intra parenchymal transducers (5–8). In addition, these devises may measure a compartmentalized local pressure (9). Some of the transducers show a low zero drift and have very low complication rates (10, 11). A non-invasive technique of monitoring ICP to replace the invasive measures mentioned would seem attractive. To be of value during NCC, it must provide a continuous and accurate bedside measure of ICP. Presently, there is no such technique available (5, 12).

### Volume Regulation of the Brain

Since the intracranial space constitutes an almost completely closed compartment, the dynamics of the ICP is determined by the physiological regulation of the contributing volumes: the CSF volume, the cerebral blood volume (CBV), and, most important, the volume of the brain tissue itself. In this section, we focus on the physiological regulation of brain tissue volume and in the subsequent section on the regulation of CBF we discus aspects on the regulation of CBV.

Like in other organs, the volume regulation of the brain is mainly determined by controlling the fluid exchange across the capillaries. Due to the blood brain barrier (BBB), the brain differs from other organs regarding these mechanisms. In addition to its other vital physiological functions, the BBB is the most important regulator of cerebral volume (13). The flux of water across a microvascular bed (*J_v_*) is described by Eq. 1:

(1) $$J_{v}=L_{p}\times A\times [\Delta P-{\sum \sigma }_{s}\times \Delta \prod_{s}]$$

*L_p_* represents the specific permeability for water (hydraulic conductivity), *A* is the surface area available for fluid exchange, Δ*P* is the transcapillary hydrostatic pressure difference, ΔΠ*_s_* the transcapillary osmotic pressure difference, and σ*_s_* the reflection coefficient of each solute (s) of the system. The product *L_p_* × *A* is denoted hydraulic conductance and reflects the total capacity for fluid exchange. Transcapillary water exchange is thus determined by the following factors: the hydraulic conductance of the capillary wall (*L_p_* × *A*), the differences in hydrostatic pressure (Δ*P*) and osmotic pressure gradient (ΔΠ*_s_*) across the capillary wall, and the endothelial component determining which solutes are reflected and will contribute to the osmotic pressure gradient (σ*_s_*).

The effective osmotic pressure across a membrane that is partly permeable to the solutes is less than the theoretical osmotic pressure. The reflection coefficient (σ*_s_*) accounts for this difference (13). The value of σ depends on the relative permeabilities of the membrane to water and to solute: if the membrane is impermeable to the solute but not to water σ equals 1, and if the permeability of the solute is identical to the diffusion coefficient in water σ equals 0. Table 1 gives the reflections coefficients of some clinically important solutes for the BBB (14). Sodium and chloride, which are the two major solutes of biological fluids, have BBB reflection coefficients of 1.0. Water passing the BBB in any direction will thus be very dilute regarding crystalloids.

**Table 1:** The reflection coefficients (see text) of various substances for the blood–brain barrier (BBB).

Solute	Reflection coefficient BBB
Urea	0.44–0.59
Glycerol	0.48
Mannitol	0.90
Sucrose	0.91–1.00
NaCl	1.00
Albumin	1.00

*Data from Fenstermacher (13) and Staverman (14)*.

**Figure 1:**
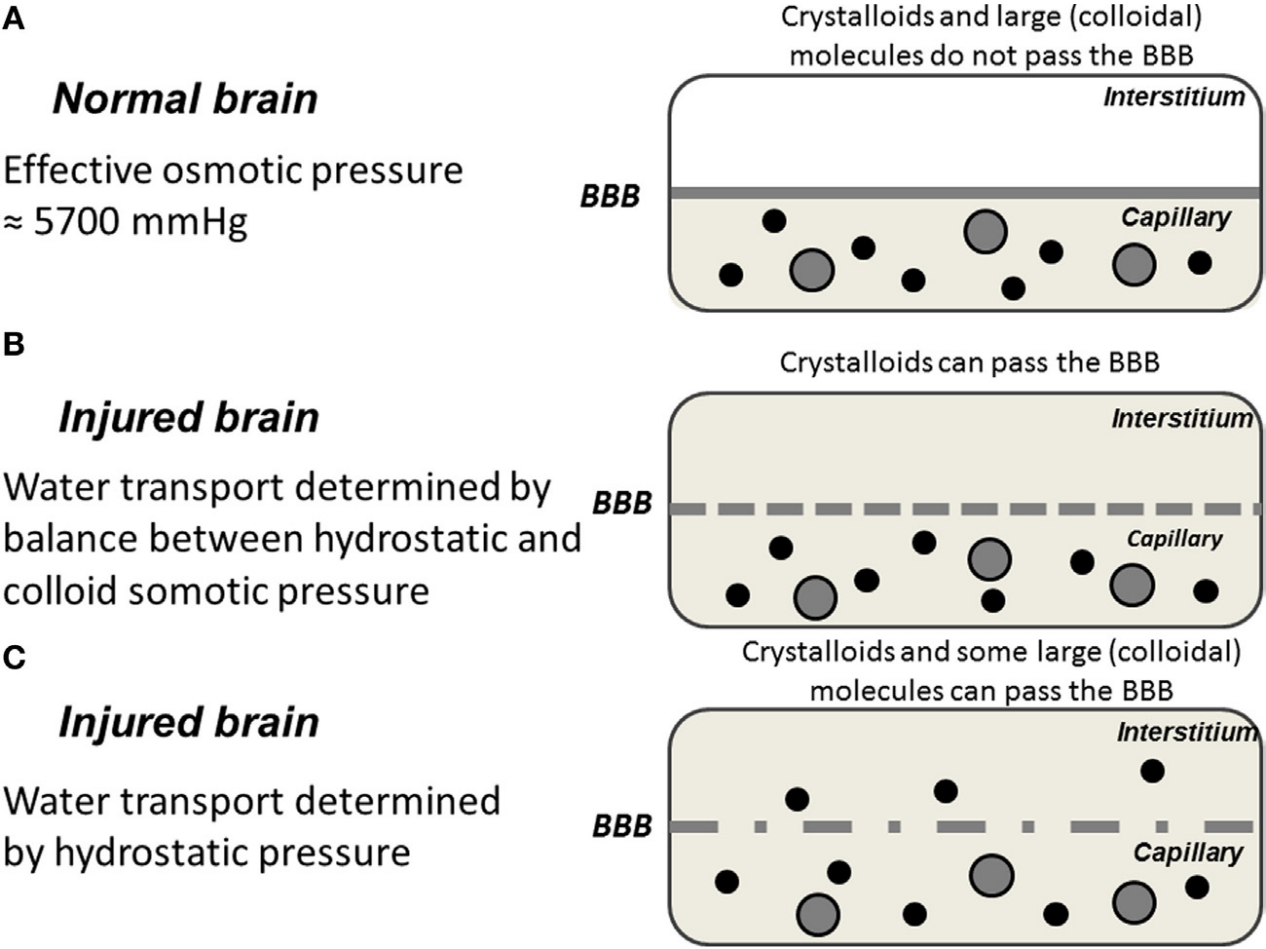
Schematic illustration of water exchange across cerebral capillaries in three hypothetical situations: **(A)** the normal brain with intact blood–brain barrier (BBB); **(B)** the injured brain with a BBB permeable for crystalloids but not colloids; **(C)** the injured brain with a ruptured BBB permeable for crystalloids as well as colloids. Gray area represents crystalloids in the capillary; black circles represent large (colloidal) molecules; filled gray circles represent blood cells.

The magnitude of the hydraulic conductance (*L_p_* × *A*) describes the rate by which water is transferred across the BBB whenever there is driving force (Δ*P* − ∑σ × ΔΠ). The only way of inducing transcapillary filtration or absorption is accordingly to affect the balance between the hydrostatic and osmotic forces across the capillary membrane. Under physiological conditions, variations in cerebral perfusion pressure (CPP; CPP = MAP − ICP) and intracerebral capillary hydrostatic pressure are of limited importance for brain volume. First, intracapillary pressure is physiologically tightly autoregulated (see Regulation of CBF) and variations in systemic blood pressure are generally not transmitted to cerebral capillaries. Second, as described above, transcapillary fluid exchange is effectively counteracted by the low permeability to crystalloids combined with their high osmotic pressure (≈5,700 mmHg) on both sides of the BBB (13). This contrasts to most other capillary regions where the osmotic pressure force is mainly derived from the difference between plasma and interstitial colloid osmotic pressure, which approximately balance the transcapillary hydrostatic pressure (≈20 mmHg). Water passing the BBB in any direction will accordingly not be accompanied by crystalloids. If, due to a difference in hydrostatic pressure, water passes the BBB an opposing osmotic gradient will immediately be created. Thus, the brain volume is under physiological conditions relatively independent of variations in intracapillary hydrostatic and colloid osmotic pressure (Figure 1). This volume control is, however, not unlimited. Although the reflection coefficient is about 1 for several solutes (Table 1) the half times for exchange of solutes like sodium and chloride indicate that a slow influx of solutes will in time dissipate the volume-controlling osmotic gradient. Accordingly, the long-term control of brain volume also depends on other mechanisms.

The lymphatic system plays an important role for volume regulation in most organs by draining interstitial fluid. Drainage of cerebral extracellular fluid has also been shown to occur *via* cranial nerves and spinal nerve roots (15, 16). This lymphatic drain has been suggested to be of importance for the immunoreactivity of the brain (16, 17) but is probably unimportant for cerebral volume regulation. The brain uses the CSF system as an alternative. The importance of this pathway for volume regulation is, however, disputed. Some studies have indicated that due to the tortuous nature of the extracellular space bulk fluid flow is negligible during physiological conditions and there are conflicting data regarding bulk flow between white matter and CSF in normal animals (18–22). In experimental brain edema, transport of extracellular fluid into the ventricular space has been described (23), but this mechanism for removing edema fluid does not solve the primary problem—the formation of edema fluid by leaky cerebral microvessels.

### Summary: ICP Monitoring and Volume Regulation of the Brain

Continuous, accurate monitoring of ICP and mean arterial pressure (MAP) is of fundamental importance for NCC. All non-surgical treatments of increased ICP are based on knowledge regarding the physiological volume regulation of the brain.

## CBF and CBF Regulation

### Principles and Techniques

A technique for quantitative, repeated measurements of CBF, which could be used under clinical conditions, was presented in the early 1960s. A radioactive tracer (^85^Kr) was administered to the brain *via* the arterial blood supply, and its clearance was registered with an extracranial detector (24, 25). Later developments of the original technique have had a major impact on our knowledge regarding CBF and the regulation of CBF during physiological and patho-physiological conditions.

Mobile CT units have been developed to be used in NCC, and Xe-enhanced CT scanning gives useful information regarding regional CBF (26). However, as the CT scanner is not CE labeled, it is presently not available in Europe. Positron emission tomography and magnetic resonance tomography techniques are giving good information of CBF but do not allow for continuous measurements and can hardly be used in NCC. Accordingly, it may be concluded that there is presently no technique available for continuous, quantitative monitoring of global or regional CBF during NCC.

Many attempts have been made to measure CBF using ultrasound Doppler methods. However, the method gives blood flow velocities which should not be interpreted as blood flow. Laser Doppler technique, mainly used in research, is available but gives relative values and the measuring volume is small. The Bowman perfusion monitor (Hemedex^®^, Hemedex Inc., Cambridge, MA, USA) is a rather new device measuring focal CBF by a thermo-dilution technique and gives blood flow in ml/min/100 g (27, 28). The device has the drawback of being sensitive for temperature changes. The device can be used for brain temperature measurements.

### Regulation of CBF

**Figure 2:**
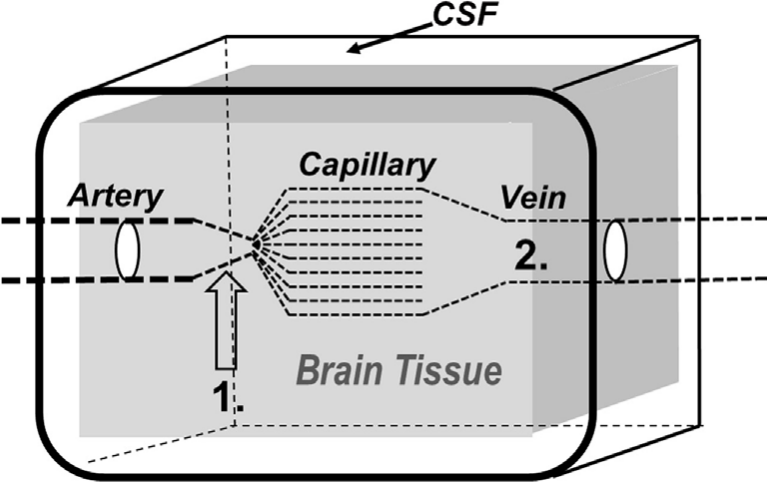
Schematic illustration of the brain and its surroundings being enclosed in a rigid shell (CSF, cerebrospinal fluid). The vessels responsible for precapillary vascular resistance (1) as well as the intracerebral venous compartment (2) are indicated in the figure.

The tight regulation of CBF secures a sufficient, continuous supply of glucose and oxygen to support the high metabolic demands of the brain (29). The control of CBF also affects two physiological parameters of importance for ICP: the intracapillary hydrostatic pressure and the intracerebral blood volume. The dynamics of ICP are closely related to three conventionally described physiological regulators of CBF: regulation by carbon dioxide (CO_2_ regulation), metabolic regulation, and pressure autoregulation. All regulators of CBF primarily act by affecting the resistance at the precapillary vessel level (Figure 2).

#### CO_2_ Regulation

**Figure 3:**
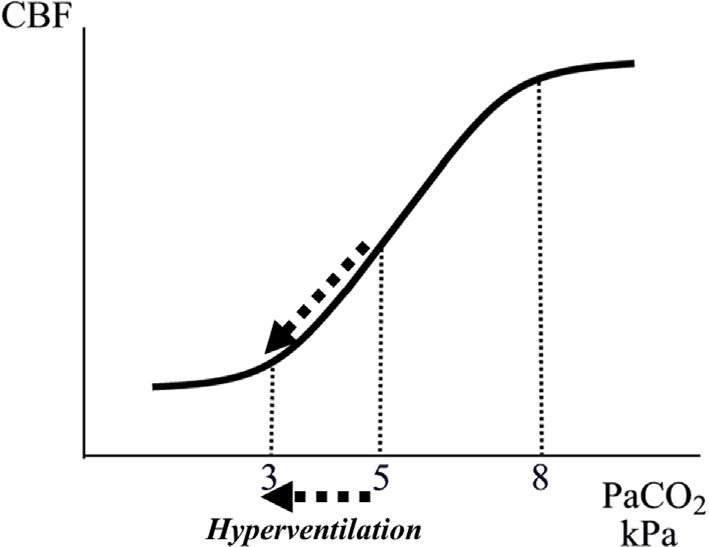
Schematic illustration of the relation between arterial tension of CO_2_ (PaCO_2_) and cerebral blood flow (CBF). The interrupted arrows indicate the decrease in CBF during a decrease in PaCO_2_ due to hyperventilation.

Controlled normal ventilation (PaCO_2_ 4.7–6.0 kPa) is currently the goal for severe traumatic brain injury (TBI) patients in the absence of cerebral herniation (30). Hyperventilation is frequently used during intensive care to achieve a rapid reduction of CBF leading to a reduction of CBV and ICP. This immediate effect (CO_2_ regulation) is caused by a pH-dependent constriction of precapillary resistance vessels (Figures 2 and 3) (29, 31). However, because the perivascular increase in pH induced by hyperventilation is compensated metabolically within a few hours, the reduction of CBF and CBV is transient despite preserved hypocapnia. Accordingly, the decrease in ICP does not last (32). Pronounced hyperventilation may also carry a risk of inducing focal ischemia (33, 34), but the clinical importance of the potential risk remains controversial (35, 36). A pro/con debate on the whether PaCO_2_ should be tightly controlled in all patients with acute brain injuries recently addressed this controversial issue (37).

Finally, as discussed later, as a result of impaired cerebrovascular CO_2_ reactivity, hyperventilation often does not reduce CBV (and ICP) in patients with very severe brain injuries (38–40).

#### Metabolic Regulation

**Figure 4:**
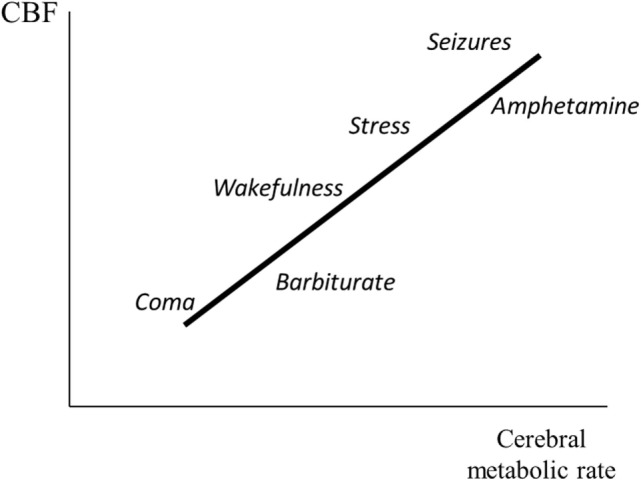
Illustration of the relation between cerebral metabolic rate and cerebral blood flow (CBF). The figure gives a schematic summary of data obtained from experimental studies during induced epileptic seizures (41, 42) and immobilization stress (43, 44), and after administration of amphetamine (45) and phenobarbitone (46).

Figure 4 schematically illustrates the relation between cerebral metabolic rate and CBF. Data are based on experimental studies of epileptic seizures (41, 42), immobilization stress (43, 44), and administration of amphetamine (45) and barbiturate (46). Cerebral metabolic rate and CBF are generally reduced during coma (29). Hypoglycemic coma is an exception. In this condition, CBF is markedly increased (47).

A reduction of cerebral energy metabolism is usually accompanied by a lasting vasoconstriction and reduction of CBF and CBV (Figure 4). The effect may be induced pharmacologically—e.g., by administration of barbiturates (39)—on condition that cerebrovascular CO_2_ reactivity is preserved (48, 49). The clinical usefulness of barbiturate coma is further reduced by the fact that prolonged high-dose barbiturate therapy is associated with pulmonary, cardiovascular, and other serious complications (50). Several other drugs that decrease cerebral energy metabolism also decrease CBF and CBV. Propofol has been used extensively for sedating patients when rapid awakening is desirable (51). However, propofol is associated with several complications and long-term propofol infusion has been related to severe adverse effects and mortality (52–55).

#### Pressure Autoregulation

**Figure 5:**
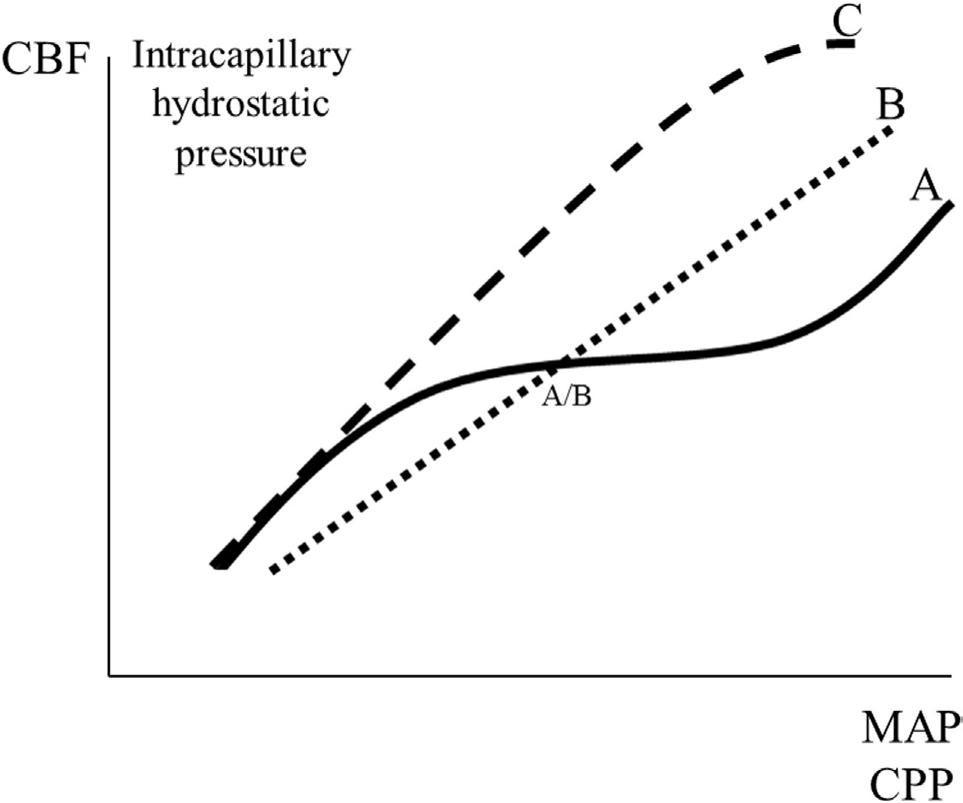
Schematic illustration of cerebral pressure autoregulation of blood flow: the relation between mean arterial blood pressure (MAP)/cerebral perfusion pressure (CPP) and cerebral blood flow (CBF)/intracapillary hydrostatic pressure. Line A indicates intact autoregulation; line C indicates absence of autoregulation; line B indicates the description of absence of autoregulation often given in neurosurgical literature. The point A/B illustrates that in neurosurgical literature intact autoregulation is often interpreted as a mechanism preventing a decrease in CBF at a decrease in MAP/CPP.

Pressure autoregulation of CBF was first described by the Danish neurologist Mogens Fog (56, 57). As a result of the pressure autoregulation, CBF remains relatively constant, despite variations in perfusion pressure within certain limits (Figure 5) (56–58). In neurosurgical literature, the physiological importance of this mechanism is often interpreted as protecting the brain from a decline in CBF (Figure 5, line B) if CPP decreases below a certain limit (AB). In physiological literature, however, autoregulation is described as a mechanism that primarily serves to keep intracapillary hydrostatic pressure constant. In this literature, if autoregulation is impaired, then blood flow is described to increase passively according to line C in Figure 5 (59, 60).

The vessels responsible for pressure autoregulation have been studied in experimental animals equipped with cranial windows for the direct observation of the cortical microcirculation (61). The large surface cerebral vessels (200–400 µm in diameter) were the most reactive to changes in blood pressure and were responsible for autoregulation between pressures of 120 and 160 mmHg. They also played the predominant role in minimizing changes in flow at pressures as low as 70–80 mmHg. The small surface and intracerebral arterioles did not change caliber when arterial pressure varied between 90 and 160 mmHg but, above 160 mmHg, they underwent forced dilation and contributed to the increase in blood flow observed.

Myogenic, metabolic, and neurogenic theories have been proposed to explain cerebral pressure autoregulation (62, 63). Additionally, endothelium-related factors have been suggested, and some studies have indicated a possible role for nitric oxide as a vasodilator during reduced CPP (64, 65). However, other studies have reported that nitric oxide may be a mediator of chemoregulation, but not autoregulation (66). Currently, it may be summarized that the mechanisms responsible for autoregulation are not completely understood and that the primary physiological function is to keep capillary hydrostatic pressure within narrow limits, despite variations in CPP. Additionally, vasodilatation may protect the brain from a fall in CBF during a moderate reduction in CPP.

For a quantitative evaluation of cerebrovascular resistance, it is necessary to measure blood flow and perfusion pressure. As there are no suitable techniques available for continuous monitoring of CBF during NCC and as autoregulation has been regarded as a clinically important phenomenon, various techniques for circumventing this problem have been presented (67). Of these, the so-called pressure reactivity index (PRx) has attracted most attention (68, 69).

Pressure reactivity index is based on continuous monitoring of the association between slow spontaneous waves in ICP and arterial blood pressure (ABP). It is calculated as a moving correlation coefficient between consecutive samples of values for ICP and ABP averaged for a defined period of time. For example, PRx may be calculated as the correlation coefficient between 30 consecutive average values over 10 s of MABP and ICP (altogether 5 min). This 5-min window may be moved forward in increments of 12 s, generating five PRx values each minute and 1-min average for PRx may be calculated and presented (68). In studies, PRx may be averaged over long-time periods (2–6 h) for comparison with subsequent CBF measurements (69).

A problem with PRx is the fact that the index is not related to a well-defined physiological mechanism or process. Accordingly, the data obtained should not be regarded as a definite measure of vascular resistance or autoregulation. For example, during the time period studied, CBF may change for many reasons not related to variations in MAP.

In several studies, PRx has been shown to correlate to clinical outcome in severe brain trauma (68, 70). It has been suggested that PRx could be used for targeting the optimal CPP (71, 72) but currently the evidence is not sufficient to make recommendations for implementing this strategy (72). Furthermore, in patients with subarachnoid hemorrhage (SAH), the interpretation of PRx was ambiguous when compared to simultaneous measures of CBF, and it was concluded that PRx was not a reliable indicator of the status of autoregulation (69).

### Summary: CBF and CBF Regulation

Presently, there is no technique available for quantitative, continuous monitoring of global or regional CBF during NCC. Three physiological mechanisms for regulation of CBF are of direct clinical importance: CO_2_-regulation, metabolic regulation, and pressure autoregulation. These basic mechanisms constitute important parts of non-surgical therapy of increased ICP.

## Cerebral Oxygenation

As normal cerebral function is completely dependent on oxidative energy metabolism bedside techniques offering possibilities of monitoring cerebral oxygenation are of obvious interest. Three of these techniques will be presented and discussed: monitoring of jugular venous oxygen saturation (SjvO_2_), brain tissue oxygen tension (PbtO_2_), and near-infrared spectroscopy (NIRS).

### Jugular Venous Saturation Monitoring

Monitoring of SjvO_2_ has been used widely in various clinical conditions: TBI, SAH, during neurosurgical procedures and NCC (73). Placement of the SjvO_2_ catheter is a relatively simple clinical routine procedure by a retrograde insertion of a jugular venous catheter. The tip of the catheter should be placed above the level of the C1/C2 disk to minimize the contamination from the facial vein (74). The choice of side is debated (75): the catheter can be placed on the side of the worst pathology, on the side where the internal jugular is most dominant, or bilaterally. Dominance may be determined by compression of each internal jugular vein separately and observation of the greater rise in ICP. Where there is no difference between the two sides, the right is commonly used as it is more likely to be the dominant side anatomically. SjvO_2_ measurements can be obtained using intermittent blood samples or continuously with the use of fiberoptic catheters that use light wavelengths in the red/infrared spectrum to calculate saturation (74). Complications may occur during catheter insertion or due to prolonged duration *in situ*. Carotid artery puncture, hematoma formation, infection, thrombosis are possible complications but reported incidence is low (74).

Given stable arterial oxygen saturation and hemoglobin concentration SjvO_2_ is interpreted as reflecting the balance between cerebral oxygen delivery (supply) and the cerebral metabolic rate of oxygen (demand). Accordingly, an increase in SjvO_2_ may occur due to hyperemia (increased supply) or decreased CMRO_2_ (decreased demand or inability to extract oxygen). A decrease in SjvO_2_ may occur due to hypoperfusion (decreased supply) or increased metabolic activity (increased demand). Therefore, SjvO_2_ may be considered as an indirect marker of CBF and cerebral metabolism (73, 76).

The normal range of SjvO_2_ remains controversial. Generally, the lower range is considered to be 50–54% whereas 75% constitute the upper range (74–78). SjvO_2_ desaturation <50% has been reported to be common following TBI associated with poorer outcome (74–78). Other clinical conditions that cause decreased SjvO_2_ include decreased systemic oxygen supply, hypoperfusion (e.g., hypotension, vasospasm, intracranial hypertension), and increased cerebral metabolism or oxygen extraction (e.g., hyperthermia, seizures) (73, 74, 79).

For obvious reasons, SjvO_2_ provides limited information in patients with focal cerebral ischemia. It has been estimated that an average of 170 ml of brain tissue was critically ischemic before SjvO_2_ levels dropped below 50% (80). Accordingly, the correlation between SjvO_2_ and PbtO_2_ is often poor and high SjvO_2_ has been reported in patients with low PbtO_2_ and focal ischemia as well as in patients near to brain death (81, 82). Furthermore, it has been reported that SjvO_2_ monitoring was useful for only 43% of the time that the catheter was *in situ*, and only half of the time that PbtO_2_ monitoring was possible (83). Discrepancies found between SjvO_2_ measurements between left and right hemisphere raise concerns also as to the choice of catheter location (75).

### Brain Tissue Oxygen Tension Monitoring

Monitoring of PbtO_2_ has been used extensively during NCC (TBI, SAH, cerebral infections) and during cerebral surgery (76, 84). The commercially available systems include Licox^®^ (Integra Neurosciences, Plainsboro, NJ, USA) and Raumedic^®^ (Raumedic AG, Helmbrechts, Germany). These probes come in different configurations, and the most advanced combines sensors for PbtO_2_, temperature, and ICP within a single catheter (Neurovent-PTO), and the Licox^®^ combines PbtO_2_ and temperature sensors. Different principles for measuring the PbtO_2_ are used in the two brands. The Raumedic^®^ brand utilizes an optical method based on quenching of luminescence. Ruthenium is used as a luminophore and oxygen is the quencher. The Licox^®^ is a polarographic electrochemical Clark-type cell electrode. This technique utilizes the electrochemical properties of noble metals to measure the surrounding oxygen partial pressure. The electrode consists of a membrane covering a layer of electrolyte and two metallic electrodes: oxygen diffuses through the membrane and is electrochemically reduced at the cathode. The greater the oxygen partial pressure, the more oxygen diffuses through the membrane. The change in voltage between the reference electrode and the measuring electrode is proportional to the amount of oxygen molecules reduced on the cathode. As the process is temperature-dependent, a temperature probe is provided with the PbtO_2_ probe to correct for variations in tissue temperature may differ in patients. The two commercially available systems have been compared during *in vitro* and *in vivo* conditions (85). Generally, the Raumedic^®^ sensors measured higher PbrO_2_ values (≈10%), but there was no significant difference regarding overall measurement or *in vitro* accuracy between the two probes.

PbtO_2_ varies with changes in arterial oxygen tension (PaO_2_) and CBF but exactly what PbtO_2_ measures remains to be defined (86–88). In the extensive clinical study by Rosenthal et al. (88), it was concluded that the product of CBF and the arteriovenous difference in oxygen tension had the strongest relationship with PbtO_2_. Several authors have, based mainly on clinical observations, suggested a threshold for PbtO_2_ below which hypoxic/ischemic cerebral damage occurs (83, 89–93). However, in an experimental study, it was concluded that the threshold values for PbtO_2_ under which energy metabolism fails was variable and most likely depending on the metabolic demands of the tissue (94). Accordingly, though PbtO_2_ may accurately describe the tension of oxygen in the tissue—which is determined by the blood flow, the blood oxygen tension, and oxygen diffusion through the tissue—but does not disclose whether this oxygen tension is sufficient for maintaining adequate metabolism or not. To answer the latter question, it is necessary to measure cerebral energy metabolism (see Microdialysis).

A relatively large number of clinical studies have suggested a relation between measured PbtO_2_ levels and mortality following TBI (83, 89–93, 95, 96). These studies have led to the hypothesis that PbtO_2_ monitoring could be used for targeting and improving intensive care in severe TBI (97–100). However, two recent publications found that PbtO_2_ guided therapy did not reduce mortality (101) and was associated with higher use of vasopressors and higher cumulative fluid balance leading to higher ICP and pulmonary edema (102).

### Near-Infrared Spectroscopy

Near-infrared spectroscopy is based on the principle that near-infrared light penetrates tissues well. About 40 years ago, the differential absorption of near-infrared light by oxyhemoglobin and deoxyhemoglobin was noted, and the technique was suggested as method to measure circulation and oxygenation in the human brain non-invasively (103). As enzymes in the mitochondrial respiration chain also have differential characteristics of light absorption depending on their redox state (in particular cytochrome c oxidase), NIRS has also been suggested for estimation of cellular metabolism (104, 105). Today, the technique of NIRS has a number of different medical applications (106).

As NIRS is a continuous, non-invasive monitor, it has been used extensively for cerebral monitoring. When the distance between incoming radiation and the reflected radiation reaching the optical sensor at the surface of the head is 30 mm, the returned radiation will pass through a depth of approximately 20 mm of tissue. Accordingly, information derived from this reflection is limited to the cerebral cortex. As the scattering coefficient of this optical path is unknown, the oxygenated/deoxygenated hemoglobin level obtained is a relative rather than an absolute value. Normal range of cerebral regional oxygen saturation (rSO_2_) has been reported to be between 60 and 75%, but individual baseline variation has been reported to be as high as 10% (107, 108). The lack of standardization among NIRS devices contributes to the difficulty of establishing definitive threshold levels. A drawback of the technique is also that there may be a portion of the oxygen content that derives from the scalp, bone, and meninges.

A number of factors may affect the NIRS signals adversely. NIRS appears to be most limited when there is preexisting brain injury. In TBI, the accuracy is often reduced by tissue swelling and the presence of extravascular blood collections in the subarachnoid, subdural, or intraparenchymal tissue compartments, the presence of subdural air after craniotomy, and a wet chamber between optical sensor and skin (109–111). Therefore, reports in TBI are conflicting. Some studies report that NIRS had a high failure rate and limited sensitivity in assessing rSO_2_ in severe TBI (110) while others have found the opposite (112, 113). Although the indication for NIRS appears to be limited within NCC, the technique is frequently used in other clinical situations when cerebral oxygenation is of interest: extracorporeal circulation during open heart surgery, status post cardiac standstill. Accordingly, neuro-intensivists should be informed about the possibilities and limitations of the technique.

### Comparison of SjvO_2_, PbtO_2_, and NIRS

The three techniques of SjvO_2_, PbtO_2_, and NIRS all permit bedside real-time, continuous data. The differences in information obtained is explained by the strengths and weaknesses of the three techniques and whether giving global (SjvO_2_) or regional (PbtO_2_, NIRS) information (83, 112, 113). Regional episodes of ischemia are accurately revealed by monitoring PbtO_2_ (81, 83) but, as it is a local technique, the accuracy is dependent on the position of the catheter in relation to focal lesions. SjvO_2_ and PbtO_2_ values correlate closely in response to changes in global parameters such as brain oxygenation and CPP (83, 114). SjvO_2_ gives information regarding global hemispheric oxygenation and may accordingly miss focal ischemia. These technical limitations should be considered when evaluating the estimation that a SjvO_2_ threshold of 50% approximately corresponds to PbtO_2_ of 8.5 mmHg (83). Correlation between NIRS and other measures of oxygenation is variable, and various results regarding such correlations have been reported (74, 78, 114). Of the three techniques, the impact of PbtO_2_ for prediction of clinical outcome has been evaluated in most detail in TBI studies. In a prospective study of 53 patients with severe TBI, the authors found that the use of both ICP and brain tissue PbtO_2_ monitors and therapy directed at brain tissue PO_2_ was associated with reduced patient death following severe TBI (115). Furthermore, in a recently published study, the authors found that reaching a critical PbtO_2_ threshold of ≤10 mmHg might be detrimental to cognitive outcomes following TBI (116).

### Summary: Cerebral Oxygenation

All three brain oxygenation monitoring techniques exhibit specific advantages and limitations. PbtO_2_ monitoring is probably currently the technique most frequently used in NCC. Monitoring cerebral oxygenation provide valuable physiological information that may help guide treatment. However, the interpretation of these data rather than the monitoring itself will determine whether the patient will benefit from such technology.

## Treatments of Elevated ICP

### Physiological Aspects on Surgical Treatments

Because the intracranial space is surrounded by the rigid skull, an increase in volume of one of the intracranial components must be at the expense of others:

(2) $$V_{\text{intracran}}=V_{\text{blood}}+V_{\text{brain}}+V_{\text{CSF}}+V_{\text{mass lesion}}$$

This basic principle behind this hypothesis was formulated more than two centuries ago when a Scottish physician, Alexander Monro (1733–1817) applied some of the principles of physics to the intracranial contents. The hypothesis was supported by experiments by a Scottish surgeon George Kellie (1720–1779). In its original form, the hypothesis had shortcomings that prompted modification by others. What finally came to be known as the Monro–Kellie doctrine, or hypothesis, is that the sum of volumes of brain, CSF, and intracranial blood is constant.

All surgical and non-surgical treatments of increased ICP aim at changing the volume of one or more of these components. The surgical treatments are directed toward three of these volumes: *V*_mass lesion_, *V*_CSF_, and *V*_intracran_. Following the initial resuscitation, early evacuation of significant focal mass lesions is the single most important treatment in patients with severe brain trauma (117, 118). CSF drainage, though it may be questioned from a theoretical point of view, can be used to reduce the amount of CSF in patients with remaining elevated ICP in spite of other measures. It is included in the recommendations by the US Brain Trauma Foundation (119). Craniectomy to increase the *V*_intracran_ is often advocated as a last resort for treatment of increased ICP (120, 121). All surgical treatments of increased ICP have one consequence in common: they all reduce *P*_tissue_. In a pathophysiological situation with increased permeability of the BBB, a decrease in *P*_tissue_ leads to increased transcapillary water transport. The consequences are known to neurosurgeons: the gradual increase in ICP always observed after evacuation of a focal lesion; the collapse of the ventricles sometimes induced by ventricular drainage; the bulging of cerebral tissue through the craniectomy. Accordingly, the rapid change in volume obtained by surgery should always be combined with non-surgical treatments aimed at a slow and lasting reduction in brain water content (122–125).

### Physiological Aspects on Non-Surgical Treatments

#### Transcapillary Absorption of Interstitial Water

From a physiological point of view, it is obvious that non-surgical treatments of increased ICP should primarily focus on transcapillary reabsorption of interstitial water. The only way of inducing transcapillary fluid absorption is by controlling the transcapillary osmotic and hydrostatic differences (see Volume Regulation of the Brain). Mannitol, urea, glycerol, and hypertonic saline are used during intensive care to decrease brain volume by osmotic withdrawal of water (cf. Table 1). The effects of mannitol infusion have been studied most widely. A rapid reduction of elevated ICP is usually obtained (126), but the effects of continuous or repeated infusions are not well documented. In experimental studies, multiple infusions of mannitol caused aggravation of cerebral edema resulting from interstitial accumulation (127). In addition to the osmotic effects, other possible benefits have been ascribed to mannitol, such as promoting CBF by decreasing blood viscosity (128) and scavenging of free radicals (129). However, no beneficial effect was obtained in a recent systematic review of mannitol therapy for increased ICP after acute ischemic stroke and cerebral parenchymal hemorrhage (130). A drawback of mannitol and urea is its shrinking effect of cells which promotes opening of the BBB.

As the intact BBB has a very low permeability for sodium chloride, intravenous infusion of hypertonic saline would be expected to decrease ICP. In experimental studies, a prompt and substantial decrease in cerebral water content has been documented after intravenous infusion of 7.5% saline solution (131), but the long-term effects are not well-documented. Several studies have shown beneficial effects of infusion of colloids in experimental brain trauma or stroke (132–136). A decrease in colloid oncotic pressure has been shown to aggravate brain edema after mild-to-moderate experimental brain trauma (137). In the “Lund Concept” presented later, preservation of the colloid osmotic pressure is obtained by albumin/plasma and red blood cell transfusions to normal albumin and hemoglobin values. In this protocol, albumin is the first choice because it is the main physiological colloidal substance of blood. Other colloids may be considered, but the use of human albumin is supported by the recent observation that albumin has a marked neuroprotective effect in experimental ischemic stroke (138).

The intracapillary hydrostatic pressure can be reduced by decreasing MAP, increasing precapillary vasoconstriction, or a combination of both. Both interventions reduce CBF, which may induce secondary injury—especially in vulnerable regions. This problem can be controlled at the bedside by monitoring regional cerebral energy metabolism. The physiological effects of reduced MAP are very different if the decrease results from reduced circulating blood volume or is caused by controlled antistress/antihypertensive treatment in a normovolemic patient (139). In the latter case, drugs (which affect peripheral circulation but do not cause intracranial vasodilatation) should be used. The combination of a β1-blocker (e.g., metoprolol) to reduce cardiac contractility and an α2-agonist (clonidine) to induce peripheral vasodilatation would be expected to be suitable, and CBF measurements in patients with severe head injury have documented that these drugs alone do not change cerebrovascular resistance (140).

#### BBB Permeability

In patients with peritumoral edema or interstitial edema surrounding a focal brain infection, corticosteroids are used to decrease BBB permeability. However, in patients with severe brain trauma, ischemic stroke, intracerebral hemorrhage, or aneurysmal SAH, large clinical trials have not shown any beneficial effects of steroid treatment (141–144), and the American Association of Neurological Surgeons (AANS) concluded that the majority of available evidence indicates that steroids do not improve outcome or lower ICP in severe TBI (145). The reasons for the discrepancy between trauma and peritumoral edema are incompletely known, but it is reasonable to assume that the molecular mechanisms are different. The mechanisms leading to BBB breakdown and edema in trauma as wells in the peritumoral area have been studied extensively and were recently summarized in two comprehensive review articles (146, 147).

Experimental studies have indicated that low-dose continuous infusion of prostacyclin might improve cerebral microcirculation and reduce capillary permeability (148, 149). Some clinical studies have indicated a possible beneficial effect of prostacyclin in severe TBI (150–153). To summarize, there is presently no therapy with documented effect for reduction of increased BBB permeability in patients with TBI or stroke. However, several novel promising strategies targeting the BBB have been presented *in vitro* and *in vivo* animal studies, though their relevance and signaling pathways in humans remain to be proven (147).

#### Intracranial Blood Volume

**Figure 6:**
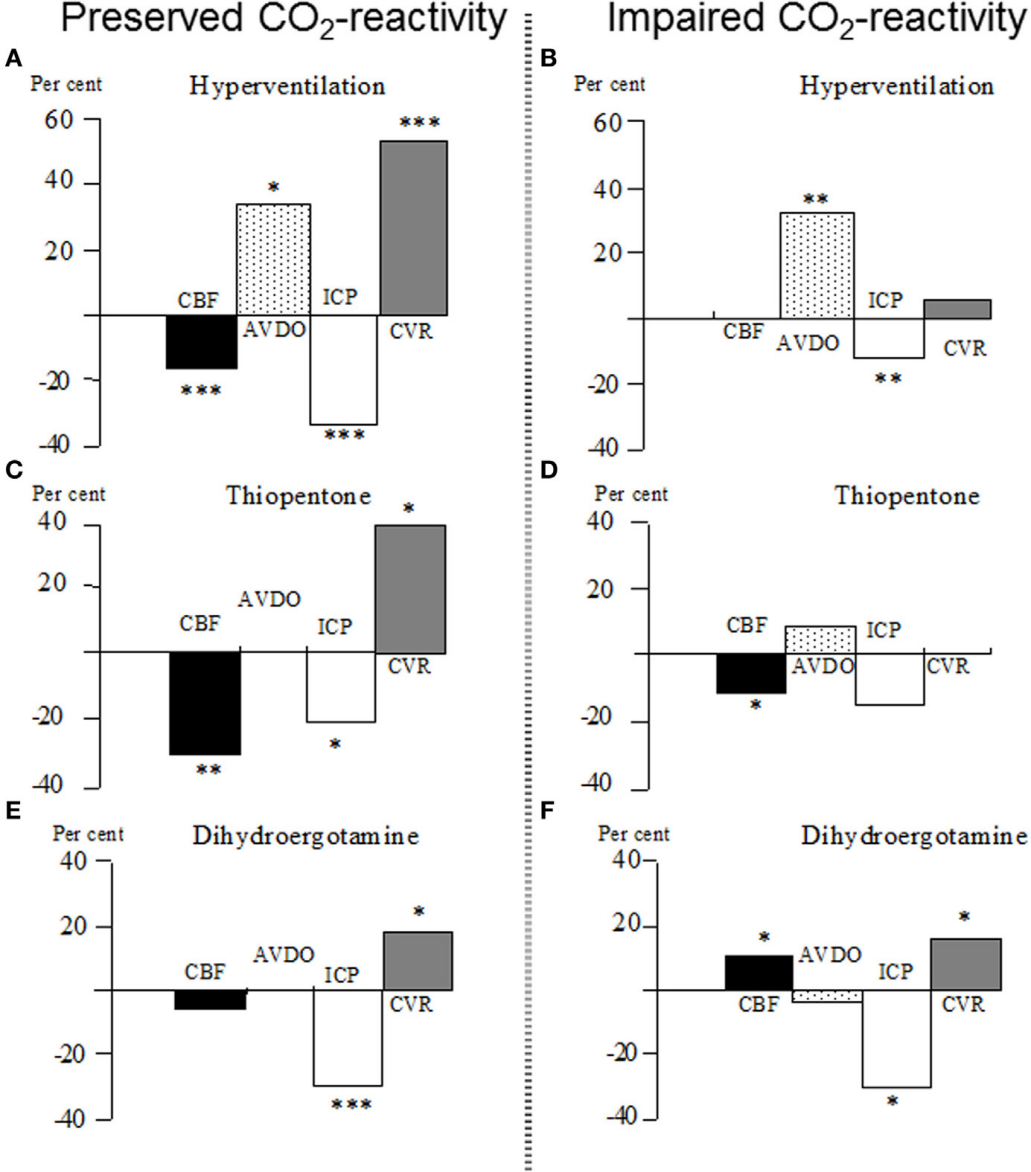
**(A–F)** Percentage changes in global cerebral blood flow (CBF), cerebral arterio-venous difference in oxygen (AVDO), intracranial pressure (ICP), and cerebro-vascular resistance (CVR) in patients with severe brain trauma. After the initial test with increased controlled ventilation (hyperventilation), the patients were assigned either to the group “Preserved CO_2_-reactivity” **(A)** or “Impaired CO_2_-reactivity” **(B)**. After restoration of normoventilation, the patients were given either a bolus of thiopentone **(C,D)** (5–11 mg/kg intravenously) or a bolus of dihydroergotamine **(E,F)** (DHE; 4 mg/kg intravenously). *p < 0.05; **p < 0.01; ***p < 0.001 for significance of difference from control value. Data from Ref. (48, 156).

Since long it has been known that ICP and intracranial blood volume is decreased during hyperventilation (154, 155). The effect is obtained by constriction of precapillary resistance vessels and always causes a reduction of CBF. Figure 6A shows the physiological effects of induced hyperventilation on global CBF, cerebral arterio-venous differences in oxygen content (AVDO), ICP, and cerebral vascular resistance (CVR) in patients with severe head injuries and preserved CO_2_ reactivity (data from 48). In patients with impaired CO_2_ reactivity (as defined by unchanged CBF), there was no change in CVR (Figure 6B) and the observed increase in AVDO and decrease in ICP were caused by regional differences in blood flow regulation not revealed by the global CBF technique (48).

The cerebral physiological effects of indomethacin are similar to those of hyperventilation, and the effects are long-lasting (157). In animal experiments, however, the reduction of ICP caused by indomethacin was always associated with signs of cerebral ischemia (158), and, consequently, the clinical usefulness of indomethacin therapy may thus be questioned. Barbiturate therapy also reduces ICP by inducing precapillary vasoconstriction. The vasoconstrictor effect obtained by barbiturate is caused by the reduction in cerebral energy metabolism. The decrease in CBF is paralleled by a reduction in energy metabolism (cf. Figure 4) and, accordingly, the vasoconstriction should not increase the risk of cerebral ischemia (48). Figure 6C illustrates that the decrease in CBF and increase in CVR are not associated with the increase in AVDO (data from 48). In patients with impaired CO_2_ reactivity, the increase in CVR and the decrease in ICP were not obtained (Figure 6D). As these patients usually also have an impaired pressure autoregulation, the minor decrease in CBF was interpreted to result from a decrease in MAP (48).

A cerebral vasoconstrictor ideal for treatment of increased ICP should have its main effect within the venous compartment and less effect on precapillary resistance (cf. Figure 2). In the peripheral circulation, dihydroergotamine (DHE) is known to preferentially constrict venous capacitance vessels (159) and DHE has been shown to decrease ICP in patients with severe head injuries (160). This effect was obtained, although CBF remained unaffected or increased and no increase in AVDO was obtained (156, 161). Figure 6E summarizes the cerebral physiological effects for patients with preserved CO_2_ reactivity (data from 156). Furthermore, as demonstrated in Figure 6F, DHE also decreased ICP in patients with impaired CO_2_ reactivity (156), and *in vitro* studies have shown that DHE has a more pronounced constrictor effect on isolated human cortical veins than arteries (162). The latter observation explains why a cerebral vasoconstrictor can cause a fall in ICP, although CBF remains unaffected or increases. As DHE is a non-specific receptor stimulator, it may be associated with severe adverse effects and complications (163, 164). Sumatriptan is a more specific 5-HT stimulator. It has been shown to decrease ICP in animal experiments (162), but thus far, it has not been used in clinical studies.

In summary, induced hyperventilation is effective for a short-term decrease of ICP but is probably unsuitable for prolonged treatment. DHE decreases the ICP, and it does not reduce CBF or increase AVDO as its main effect is exerted on the venous compartment. However, DHE may cause severe side effects and complications. The ideal pharmacological vasoconstrictor remains to be defined but presently reduction of cerebral energy metabolism (usually obtained by infusion of a low-dose barbiturate) is the first choice.

#### The “Lund Concept” Therapy of Increased ICP

The “Lund concept” was originally developed to treat patients with severe brain trauma, impaired CO_2_ reactivity, and a dangerous increase in ICP (122). In these patients, conventional therapies of increased ICP appeared to be ineffective (40, 48). The protocol has later been used in all patients with severe traumatic brain lesions as well as in patients with other cerebral disorders with increased ICP. For obvious reasons, the “Lund Concept” is not used in conditions with compromised arterial blood flow (e.g., arterial vasospasm after SAH).

In patients with head injury, the therapy begins after initial resuscitation and surgical evacuation of focal mass lesions. The patients are treated with controlled normoventilation (without muscle relaxation) under continuous monitoring of MAP and ICP. In 1995, the technique of intracerebral microdialysis and bedside biochemical analysis of cerebral energy metabolism was introduced to document that the therapy did not cause a dangerous decrease in CPP. The protocol followed in the “Lund Concept” is based on the physiological considerations mentioned earlier and can be summarized as follows.

##### Reduction of Stress Response and Cerebral Energy Metabolism

Stress response is initially reduced by liberal use of sedatives (benzodiazepines). A further reduction of the stress response and catecholamine release is obtained by a continuous infusion of low-dose thiopental (0.5–3 mg/kg/h) and fentanyl (2–5 μg/kg/h). The dose of thiopental is kept low to avoid cardiac inhibition, pulmonary complications, and other side effects (50). A protective effect of β1-blockade after head injury, through reduction of sympathetic nervous system effects on the heart and lungs have been suggested. Reports of the beneficial effect of β-blockade in head injury with regard to survival have been published (165–168).

##### Reduction of Capillary Hydrostatic Pressure

Mean arterial pressure is reduced to the physiological level for the age of the individual patient with a combination of the β1-antagonist metoprolol (0.2–0.3 mg/kg/day, intravenously or as a low continuous intravenous infusion) and the α2-agonist clonidine (0.4–0.8 μg/kg, 4–6 times/day, or a low dose of continuous, intravenous infusion) (125, 140). The antihypertensive treatment is initiated after evacuation of focal mass lesions when the patients are clearly normovolemic (as obtained by red blood cell and albumin/plasma transfusions to normal albumin and hemoglobin values and to a normal central venous pressure). A CPP of 60–70 mmHg is considered optimal; however, if necessary, a transient decrease in CPP (to 50 mmHg in adults and 40 mmHg for children) is accepted to control ICP (122).

##### Maintenance of Colloid Osmotic Pressure and Control of Fluid Balance

Red blood cell transfusions and albumin are administered to achieve normal values (Hemoglobin: 125–140 g/l, Serum-albumin: approximately 40 g/l) to ensure normovolemia and to optimize oxygen supply. The albumin/plasma/blood transfusions also serve the purpose of obtaining a normal colloid osmotic pressure, favoring transcapillary absorption. A balanced or moderately negative fluid balance is a part of the treatment protocol and is achieved by diuretics (furosemide) and albumin infusion. All patients are given a low-calorie enteral nutrition (max energy supply: 15–20 kcal/kg/day). These physiological principles followed during NCC are supported by experimental studies (123, 125, 169–171).

##### Reduction of CBV

Intracranial blood volume may be reduced both on the arterial side with thiopental (48) and DHE (159–161) and on the venous side with DHE (156, 161). However, due to the serious complications related to DHE therapy (163, 164), this treatment is rarely used.

#### The “Vasoconstrictor Cascade” Therapy of Increased ICP

The “Lund Concept,” advocating a reduction in CPP, was first presented in 1994 (125). In 1995, a contradictory protocol based on physiological considerations and advocating a pharmacologically induced increase in CPP as therapy for increase in ICP was published (172). The therapeutic principle was based on the so-called “Vasoconstrictor Cascade.”

**Figure 7:**
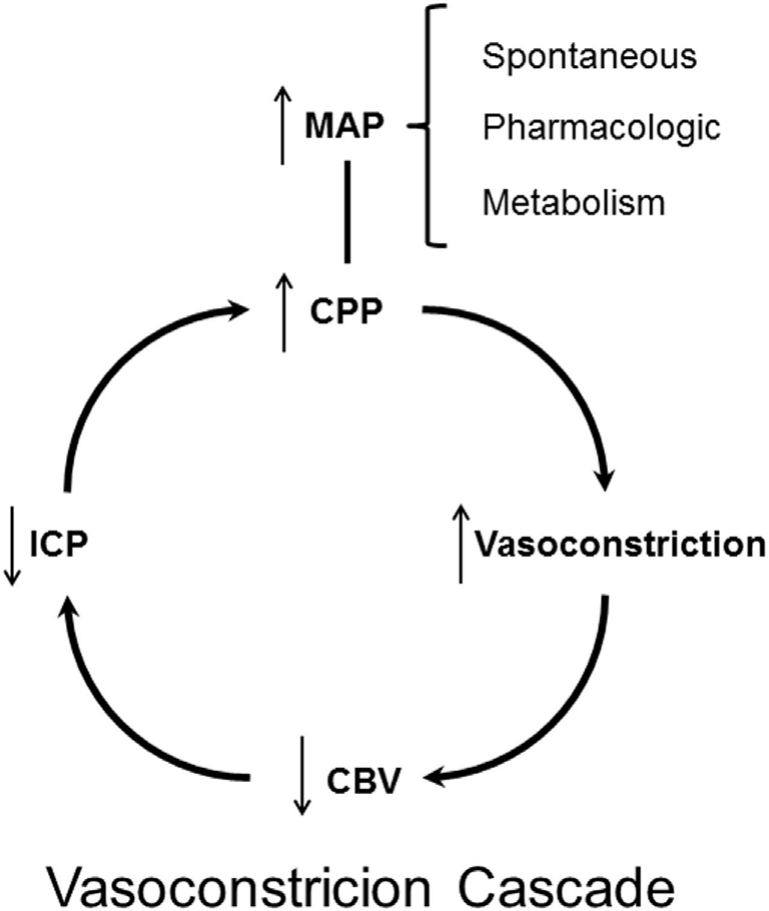
Schematic illustration of the theoretical principles behind the “Vasoconstrictor Cascade” according to Rosner et al. (172).

The theory was based on the hypothesis that in patients with a functioning pressure autoregulation an increase in MAP would, due to the induced vasoconstriction, cause a decrease in CBV and hence also a decrease in ICP (Figure 7). In patients with supposed impaired pressure autoregulation, it was assumed that the autoregulation curve was often shifted to a higher MAP level. By this mechanism, the authors postulated that marked increase in MAP from a vigorous use of vasopressors would result in a decrease in CBV and ICP: “Cerebral perfusion pressure management can serve as the primary goal in the treatment of traumatic intracranial hypertension with substantially improved mortality and morbidity following TBI. The minimum level of CPP in this instance is greater than 70 mm Hg and frequently higher, defined by individual circumstances that may occasionally require a level of 100 mm Hg or more, but average 85 mm Hg. Systemic hypertension and iatrogenic maintenance of CPP do not potentiate or worsen intracranial hypertension” (172).

Does the “Vasoconstrictor Cascade” comply with accepted physiological principles? By definition pressure autoregulation implies that CBF remains constant. CBF is controlled by the constriction/dilatation of the precapillary resistance vessels (Figure 2). As stated above, all surgical and non-surgical treatments of increased ICP act by changing the volume of one or more of these components. Assuming that the total intracranial volume is 1,500 ml (*V*_blood_ ≈75 ml) the distribution of the volumes of blood are approximately *V*_venous_ 50–55 ml, *V*_arterial_ 10–20 ml, *V*_capillaries_ 5–10 ml. Probably, the vessels responsible for autoregulation are small pial arteries with diameters between 200 and 400 µm (cf. above CBF, autoregulation). A decrease in diameter of these small vessels would accordingly cause a minor decrease in intracranial blood volume. However, by definition pressure autoregulation implies that CBF remains constant and, as discussed above, the decrease in CBV and ICP caused by constriction of precapillary resistance vessels occur at the expense of a reduction of CBF. Furthermore, according to the Poiseuille–Hagen equation, the flow through a vessel is proportionate to the radius to the fourth (*R*^4^), while the volume is proportionate to *R*^2^. Accordingly, it seems unlikely that preserved capacity for autoregulation and pharmacological increase in MAP could be useful for treatment of increased ICP. When tested in acutely head injured patients, it was documented that vasopressor therapy resulted in a marked increase in ICP in the group with severe brain trauma (126).

### Summary: Treatments of Elevated ICP

All treatments of elevated ICP act by reducing one or more of the intracranial volumes (or increasing the total available volume through craniectomy). Non-surgical treatments act by decreasing brain and/or blood volume. The “Lund concept” is based on the physiological principles for regulation of these volumes. Many of these aspects are now included in protocols for treatment of increased CBF. However, it is still motivated to mention that the common belief that ICP may be decreased by an induced increase in MAP (“Vasoconstrictor cascade”) is not supported by known physiological principles.

## Cerebral Energy Metabolism

The human brain constitutes only about 2% of the body weight but approximately 20% of the total body energy is consumed by the brain. To cover this very high energy demand, CBF constitutes 15% of cardiac output. The majority of the energy is used for active transport of various compounds against their concentration gradients, and it is estimated that less than 20% of the energy utilized by the brain is used for biosynthesis of cellular components. Due to the fact that glucose is the only substrate that is transported across the BBB at sufficient speed, glucose is under normal circumstances the sole substrate utilized by the brain. In the cytoplasm of the cells, glucose is converted to pyruvate (and lactate). The main part of the energy is obtained after pyruvate has entered into the citrate cycle in the mitochondria, ultimately being completely degraded to carbon dioxide and water. Global cerebral energy metabolism can be evaluated by measuring CBF and arterial venous difference in oxygen content. These basic physiological and biochemical principles of cerebral energy metabolism have been known for decades and were described in a textbook in 1978 (29). However, as pathophysiological processes are often focal global, techniques for the evaluation of metabolism may give information of limited importance during NCC. Furthermore, it would be of clinical importance to monitor cerebral energy metabolism and indicators of cellular damage bedside. Intracerebral microdialysis is presently the only technique that offers this possibility.

### Microdialysis

The microdialysis technique was developed more than 30 years ago for monitoring chemical events in the animal brain (173, 174) and has become an accepted scientific standard technique. Altogether, there have been well above 16,000 published studies utilizing microdialysis. In the late 1980s, the possibilities for monitoring the human brain were first explored (175–177), and microdialysis has since then been used for biochemical monitoring of most human tissues. In 1995, CMA Microdialysis (Stockholm, Sweden; present manufacturer M Dialysis, Stockholm, Sweden) introduced a sterile microdialysis catheter, a simple microdialysis pump and a bedside biochemical analyzer. The instrumentation was originally intended for subcutaneous and intramuscular use. With a slight modification of the microdialysis catheter, it has been used intracerebrally as an integrated part of routine multi-modality monitoring since 1996.

#### The Microdialysis Technique

The original idea of microdialysis was to mimic the function of a blood capillary by inserting a thin dialysis tube in the tissue to analyze the chemical composition of the interstitial fluid. However, though the microdialysis catheter is thin (0.6 mm), it is much wider than a capillary and far bigger than the estimated intercellular distance (about 0.0001 mm). The wall of the catheter allows free diffusion of water and solutes between the surrounding interstitial fluid and the perfused solution (perfusate). The concentration gradients between the interstitial fluid and the perfusate constitute the driving force for diffusion. The molecular weight of the molecules being sampled is limited by the pore size of the dialysis membrane (cutoff). The perfusate flows along the dialysis membrane slowly and at a constant speed, and the sample (dialyzate) is collected in microvials and analyzed biochemically bedside by utilizing enzymatic, colorimetric techniques.

The achieved concentration of the analytes in the dialyzate depends on the degree of equilibration between the interstitial fluid and the perfusate. This relation is termed (relative) recovery and is defined as the dialyzate/interstitial concentration ratio expressed as percentage: Recovery = Conc_dialyzate_/Conc_tissue_.

Accordingly, the microdialysis technique does usually not give the absolute concentration of the studied biochemical variables. When clinical microdialysis is performed as a routine technique, this limitation is without significance. However, some of the factors determining the recovery are important to recognize.

The three most important factors affecting recovery *in vivo* are the area of the semi-permeable membrane (length of microdialysis membrane), the perfusion flow rate, and the diffusion in the surrounding interstitial fluid. The recovery increases in proportion to the length of the dialysis membrane area. The 70 Brain MD Catheter routinely used in the brain has a membrane length of 10 mm. The diameter of the probe is about 0.6 mm, and the standard cutoff of the dialysis membrane (during clinical routine) is 20 kDa. For special scientific purpose (monitoring of large molecules, e.g., cytokines), microdialysis catheters with 100 kDa cutoff are commercially available (178). The standard perfusion flow rate used during clinical routine is 0.3 µl/min, which allows sampling for bedside analysis every 30 min. Due to the slow perfusion rate and the large dialysis membranes, recovery is high: the *in vivo* recovery for the intracerebral 70 Brain MD Catheter is approximately 70% for the biochemical variables used routinely (179). If the perfusion rate is increased to permit more frequent sampling, recovery decreases to about 30% at 1 µl/min (179).

The diffusion rate in the surrounding interstitial space is of importance and varies with the molecular weight of the studied analytes and size and tortuousity of the interstitium. The recovery may thus vary between tissues and changes with the pathophysiological conditions. The problem is unimportant for clinical routine but is very relevant, for example when microdialysis is used for quantitative pharmacokinetic studies (180, 181). The importance of the diffusion limitation of the surrounding interstitial space also explains why it is useless to perform *in vitro* calibration to compensate for the recovery *in vivo*.

#### Biochemical Variables Monitored during Clinical Routine

**Figure 8:**
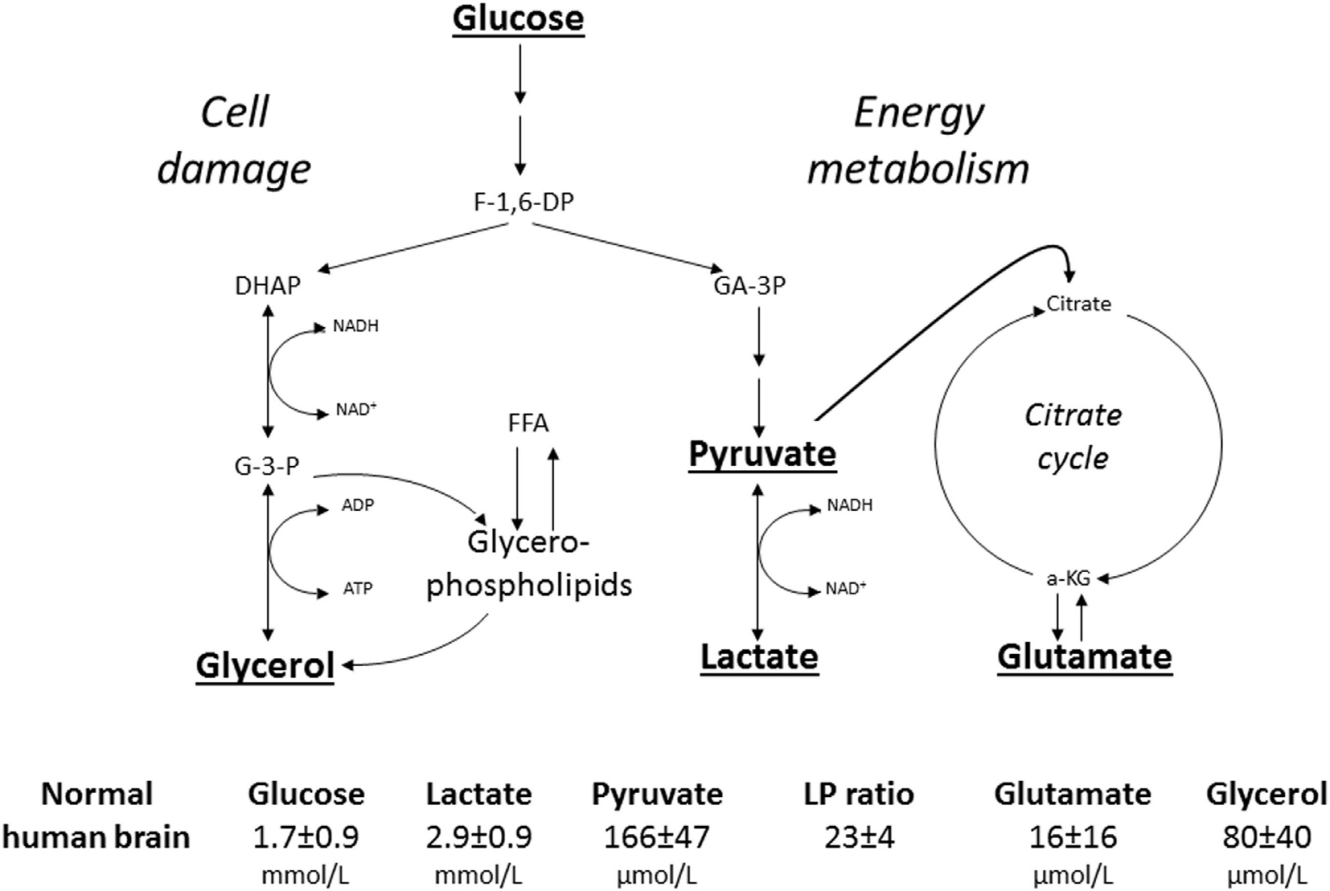
Schematic diagram of cerebral intermediary metabolism, with a focus on the glycolytic chain and its relation to glycerol and glycerophospholipids and to the citric acid cycle (Krebs cycle). F-1,6-DP: fructose-1,6-diposphate; DHAP, dihydroxyacetone-phosphate; GA-3P, glyceraldehyde-3-phosphate; G-3-P, glycerol-3-phosphate; FFA, free fatty acids; α-KG, α-ketoglutarate. Underlined metabolites are measured at the bedside with enzymatic techniques. References levels of the various metabolites for normal human brain obtained from Ref. (182).

The biochemical variables used for routine monitoring during clinical conditions were chosen to cover important aspects of cerebral energy metabolism and to give indications of degradation of cellular membranes. Figure 8 shows these variables and their reference levels as obtained in normal human brain (182). Under normal conditions, glucose is the sole substrate for cerebral energy metabolism. In the cytosol, it is degraded to pyruvate (glycolysis) with a net yield of two ATP for each molecule of glucose. Due to the redox conditions, part of the pyruvate is converted to lactate. The lactate/pyruvate (LP) ratio reflects cytoplasmatic redoxstate, which can be expressed in terms of the lactate dehydrogenase equilibrium:

(3) $$\frac{\left[\text{NADH}\right]\left[{\text{H}}^{+}\right]}{\left[{\text{NAD}}^{\text{+}}\right]}=\frac{\left[\text{Lactate}\right]}{\left[\text{Pyruvate}\right]}\times K_{\text{LDH}}$$

Thus, the LP ratio gives information regarding the efficacy of cerebral oxidative energy metabolism. The major part of pyruvate enters the citric acid cycle in the mitochondria and is completely degraded to CO_2_ and H_2_O with a net yield of another 36 ATP. The chemical relation between the citric acid cycle and the excitatory transmitter glutamate is shown in Figure 8. However, the glutamate level obtained by microdialysis does not exclusively reflect liberation of the transmitter. As interstitial glutamate is normally rapidly taken up by the astrocytes against a concentration gradient (183), an increase in interstitial glutamate is often an indicator of jeopardized energy metabolism and release from leaky cells (184).

Since the brain does not contain any triglycerides (TG), a high level of intracerebral glycerol is considered to be a reliable indicator of degradation of the glyceropospholipids of cellular membranes and cell damage (185, 186). In other tissues, and in particular in fat tissue, glycerol is mainly obtained from degradation of TG. Lipolysis is under sympathetic control through catecholamine receptors on the adipocytes, which are stimulated by circulating catecholamines as well as by local noradrenergic nerve endings. The glycerol level in subcutaneous fat tissue may be used as an indicator of physical as well as mental stress (187).

During clinical routine, the biochemical variables may be analyzed bedside (ISCUSflex, MDialysis, Stockholm, Sweden). An analytical validation of the enzymatic techniques used for analysis of glucose, lactate, and pyruvate has recently been presented (188). For critical threshold, intra- and interassay coefficients of variation (CV) were, respectively, 3.1 and 4.5% for glucose, 3.5 and 4% for lactate, and 3.3 and 4.3% for pyruvate and inter-assay CV for LP ratio was 5.9%. The data prove that these routine analyses have the accuracy and precision required for clinical application in neurointensive care but the CV must be considered when these analytical techniques are used for scientific purpose (189).

#### Intracerebral Microdialysis in NCC

**Figure 9:**
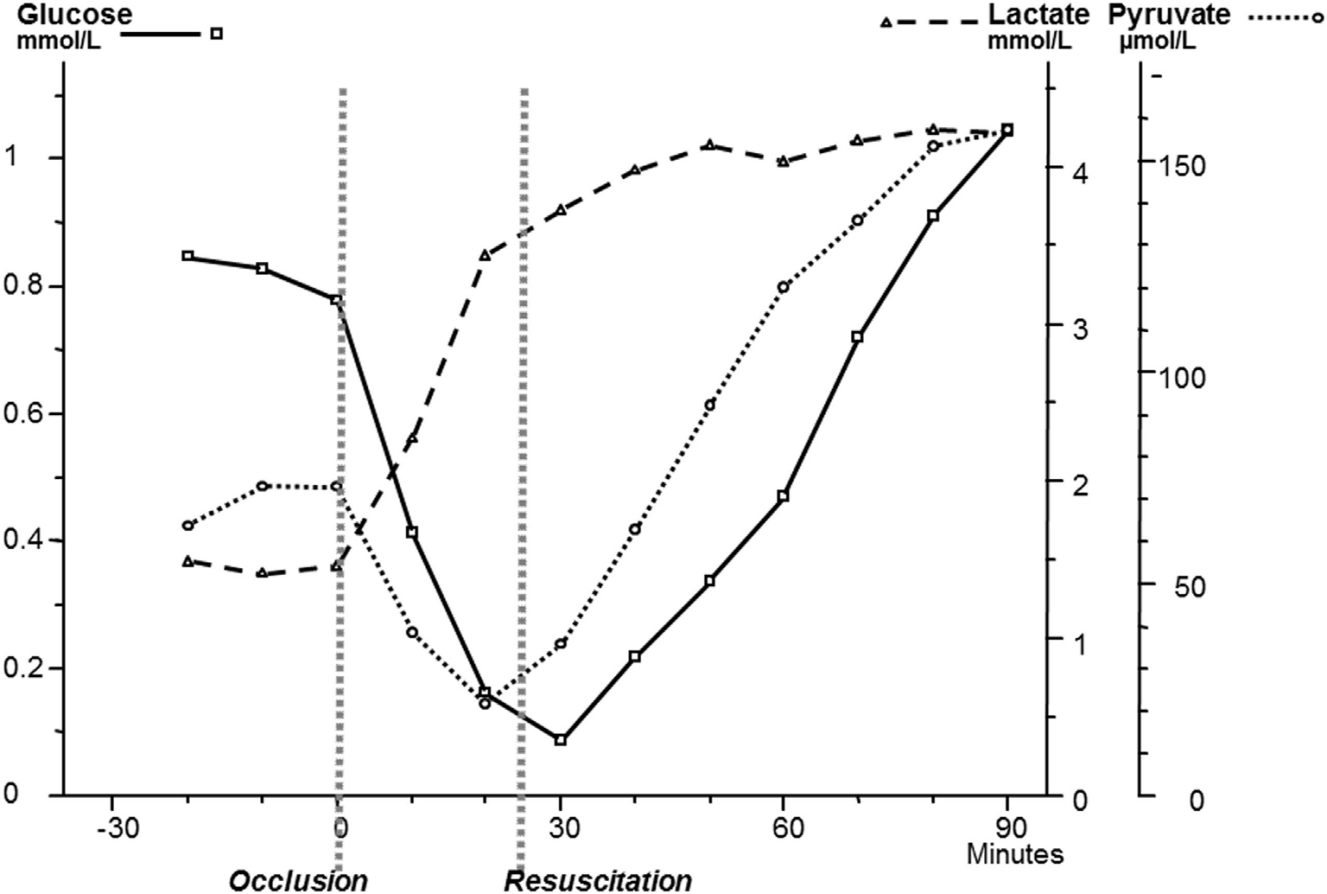
Changes in intracerebral biochemistry during transient global cerebral ischemia. Levels of glucose, lactate, and pyruvate (mean ± SD). Data from Ref. (190).

**Figure 10:**
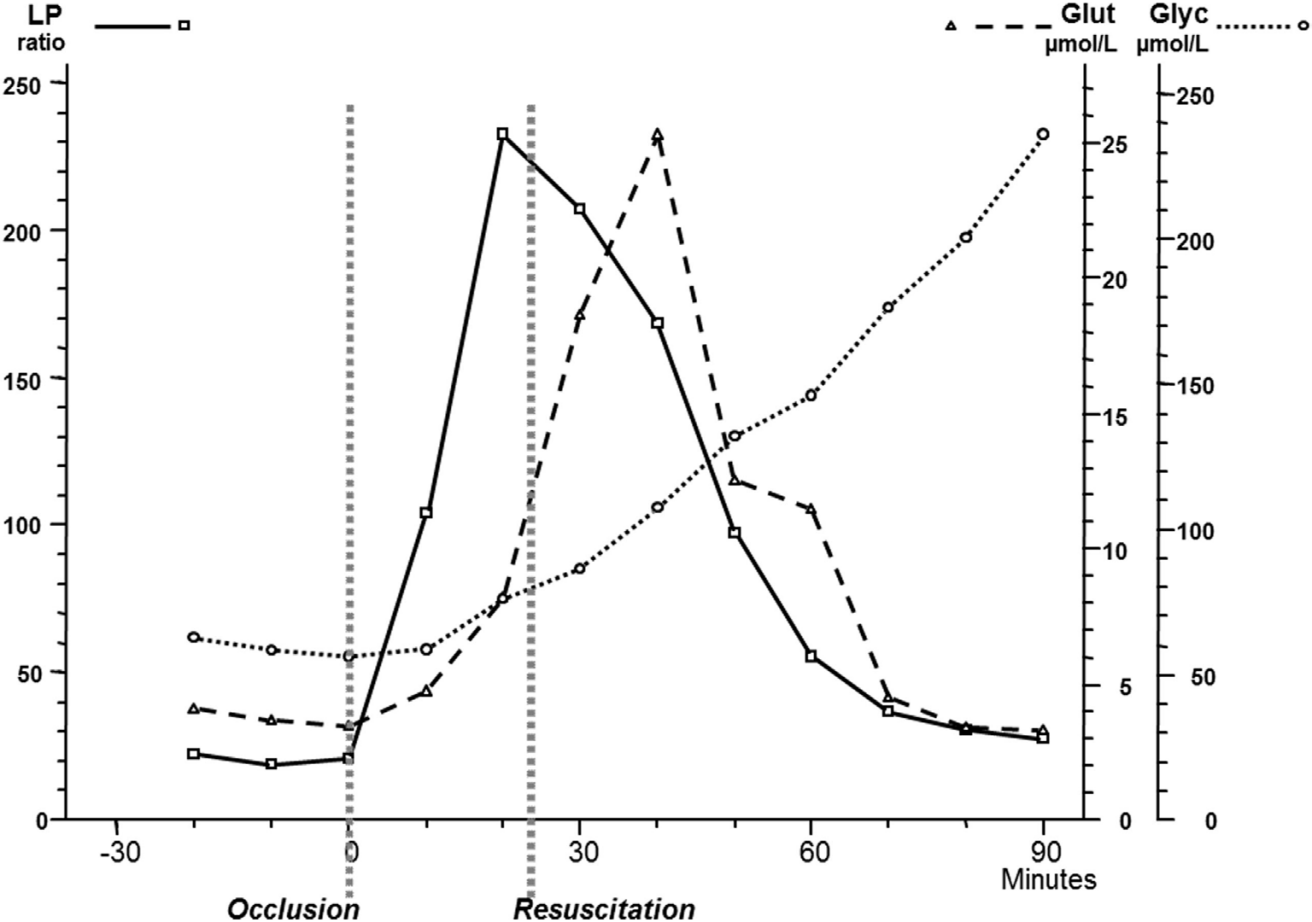
Changes in intracerebral biochemistry during transient global cerebral ischemia. Levels of lactate/pyruvate (LP) ratio, glutamate, and glycerol (mean ± SD). Data from Ref. (190).

Though microdialysis has been used in most human tissues, the majority of clinical studies utilizing microdialysis have been performed in the brain. The biochemical variables routinely analyzed bedside were chosen to cover important aspects of cerebral energy metabolism (glucose, pyruvate, and lactate), to indicate excessive interstitial levels of excitatory transmitter substance (glutamate) and to give indications of degradation of cellular membranes (glycerol) (Figure 8). Most basic principles regarding cerebral energy have been known since decades (29) and similar patterns of changes have been described when utilizing intracerebral microdialysis. Figure 9 shows changes in the intracerebral levels of glucose, lactate, and pyruvate after induction of cerebral ischemia, and in Figure 10, the simultaneous changes in LP ratio in the levels of glutamate and glycerol are shown (190). In this study, transient brain ischemia was induced in fetal lambs *in utero* by occlusion of the umbilical cord followed by resuscitation after cardiac standstill. The microdialysis technique was identical to that used during clinical conditions, but the perfusion rate was increased (1.0 µl/min) to allow frequent sampling. Induction of ischemia caused an almost instantaneous increase in the LP ratio shortly afterward followed by an increase of the glutamate level. Glucose, pyruvate, and glutamate rapidly recovered after resuscitation, but the levels of lactate and glycerol continued to be elevated and the LP ratio remained slightly above the pre-ischemic level.

These data are of importance for the interpretation of our clinical findings. The LP ratio, reflecting the redox state of the cytoplasm, will increase immediately when delivery of oxygen is insufficient and will rapidly return close to normal upon re-oxygenation. The lactate level rapidly increases during ischemia but remains elevated when circulation is restituted. Glycerol, the indicator of degradation of cellular membranes, increases relatively slowly during energy failure and remains elevated for some time when energy metabolism is normalized. The interstitial glucose level, finally, reflects the balance between delivery from the blood capillaries and the cellular uptake.

It is important to realize that the microdialysis technique gives biochemical information only concerning a small volume surrounding the catheter since regional differences in blood flow and energy metabolism are considerable in most pathophysiological conditions. The fact that microdialysis is a regional technique may thus be regarded as an advantage provided that the positioning of the catheters can be visualized in relation to the focal injuries. Furthermore, one must be aware of the dynamics of the tissue condition which may considerable change with time (191). Thus, if the tissue targeted at catheter insertion was the “worst site,” it may be less so after a while due to the evolution of tissue damage.

It has been possible to identify the metabolic pattern in various parts of the injured brain by inserting multiple intracerebral microdialysis catheters in patients with severe traumatic brain lesions. The studies have shown that “biochemical penumbra zones” surround focal brain lesions and that most adverse secondary events primarily affect these sensitive zones (192, 193). Thus, intracerebral microdialysis with bedside biochemical analysis may be used to detect and treat focal adverse events before they have caused cellular degradation or clinical deterioration that may be detected by conventional monitoring.

**Figure 11:**
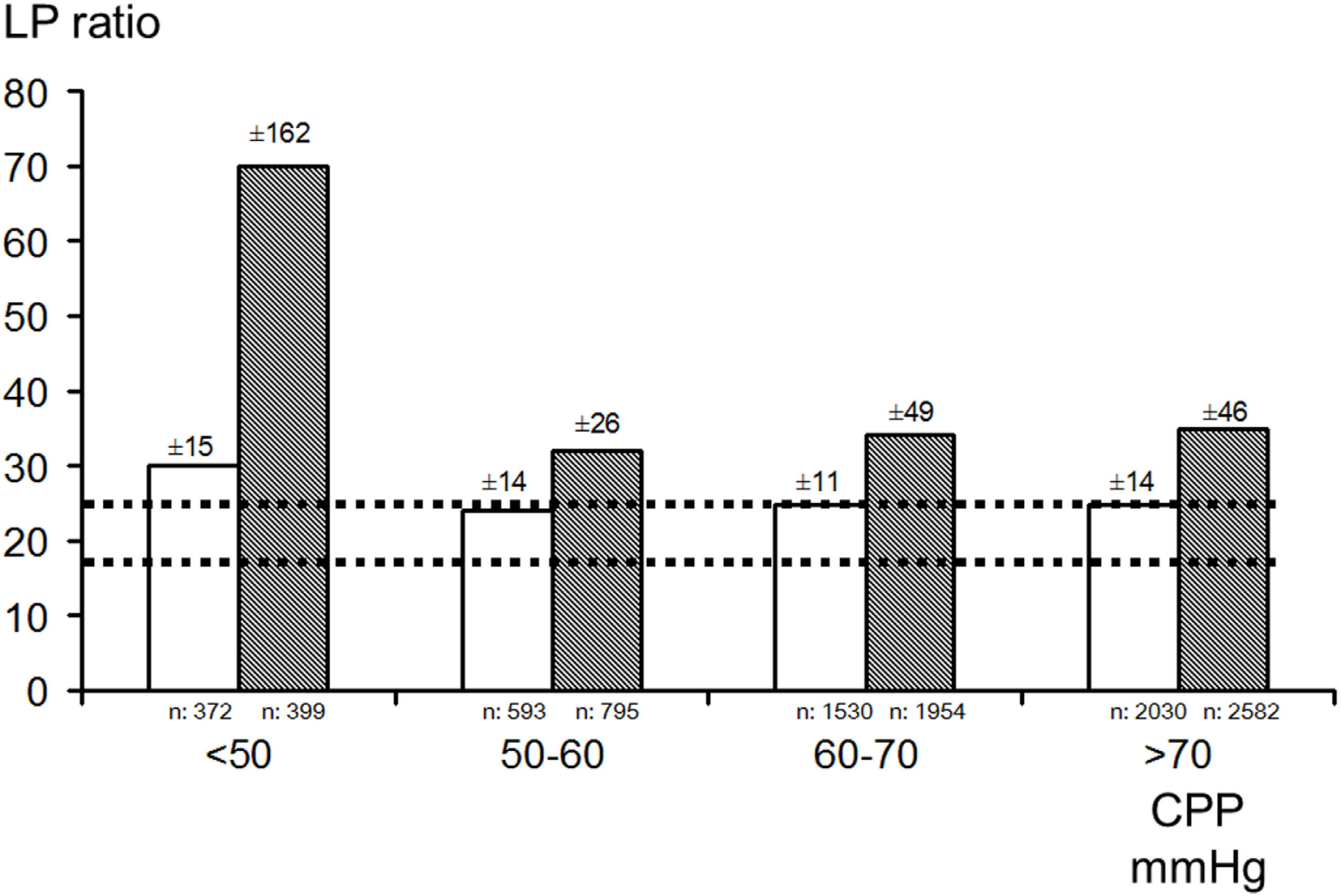
Bar graphs demonstrating mean (±SD) lactate/pyruvate (LP) ratio in the better (white bar) and worse (gray bar) microdialysis catheter positions in relation to four ranges of cerebral perfusion pressure (CPP) in patients with severe traumatic brain lesions. Interrupted lines indicate the range (mean ± SD) in healthy brains in humans during wakefulness. Data from Ref. (182, 194), respectively.

Intracerebral microdialysis has been used to determine the lower acceptable limit for CPP in the individual patient (194, 195) (Figure 11). As discussed previously, this level is of particular importance in patients with increased ICP partly due to brain edema. In these patients, a high CPP may increase the intracapillary hydrostatic pressure and cause a net transport of water into the brain tissue interstitium which will further increase ICP (see Volume Regulation of the Brain).

### Bedside Diagnosis of Cerebral Ischemia and Mitochondrial Dysfunction

During NCC, microdialysis has primarily been focused on identifying episodes of secondary clinical ischemia. However, several studies have documented that during NCC prolonged disturbance of cerebral energy metabolism and increase of LP ratio were often not due to ischemia as cerebral oxygenation remained unaffected (196). Already in the 1970s, animal experiments had shown that transient cerebral ischemia often lead to a prolonged period of mitochondrial dysfunction (197, 198).

**Figure 12:**
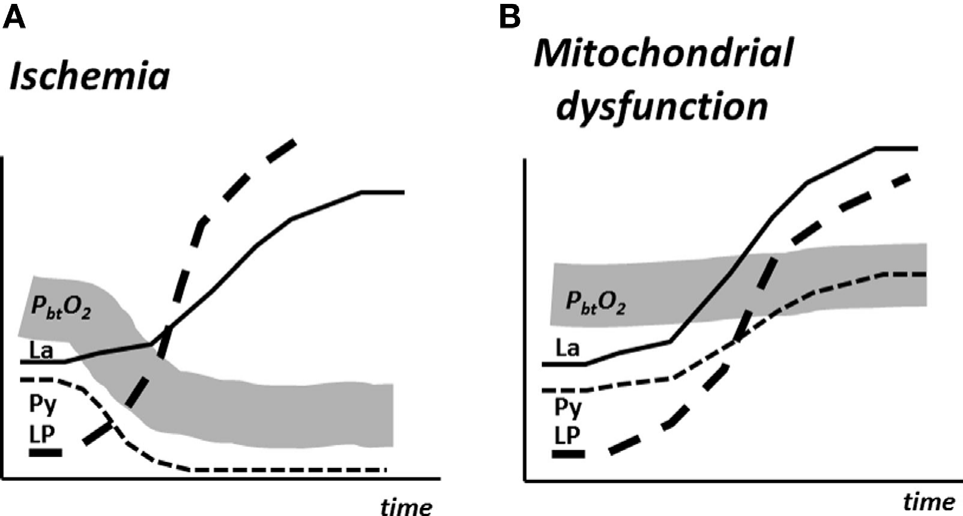
Schematic illustration of cerebral tissue oxygenation (P_bt_O_2_) and changes in the levels of lactate (La), pyruvate (Py), and the lactate/pyruvate (LP) ratio in experimental cerebral ischemia **(A)** and mitochondrial dysfunction **(B)**. Data from Ref. (200).

The biochemical pattern obtained during mitochondrial dysfunction has been described recently (199, 200). The results are shown schematically in Figure 12 and compared with the corresponding metabolic pattern in cerebral ischemia. In cerebral ischemia, the interruption of blood flow and decrease in P_bt_O_2_ causes a very rapid increase in LP ratio (Figures 9 and 12A). As the cerebral delivery of substrate for energy metabolism (glucose) is also interrupted, pyruvate decreases to a very low level and, as a result, the LP ratio increases to extremely high levels. In mitochondrial dysfunction, P_bt_O_2_ is unchanged but, due to impaired mitochondrial function, oxidative metabolism is insufficient to meet the energy demands. The increase in glycolytic rate causes a massive production of lactate and increase in the LP ratio although tissue pyruvate remains at a normal level or increases slightly (Figure 12B). Under clinical conditions, an increase in LP ratio may be caused by a variety of mechanisms (201). Drugs that are effective in mitochondrial dysfunction are presently under investigation. One example is cyclosporine A, which is thought to decrease mitochondrial damage by blocking opening of the mitochondrial permeability transition pore (202). The protective effect of cyclosporine in cerebral ischemia was in 1995 described in experimental studies (203). However, its clinical usefulness has not yet been documented. Irrespective of the mechanisms underlying mitochondrial dysfunction, a beneficial therapeutic intervention would probably be reflected in normalization of the biochemical variables analyzed and displayed at the bedside.

### Lactate Supplementation for Treatment of TBI Patients

Based on association with the so-called astrocyte-to-neuron lactate (ANL) shuttling hypothesis, a number of publications have suggested the use of lactate as supplemental brain fuel after TBI. The ANL shuttle model was based on an observed glutamate-evoked release of lactate in cultured astrocytes indicating that glycolysis provides the ATP needed for astrocytic uptake of neurotransmitter glutamate and its conversion to glutamine and the assumption that the released lactate is oxidized by nearby neurons (204). However, ANL shuttling has never been quantified and validated as being metabolically significant in living brain, the cellular source(s) of lactate in brain have not been identified, and there are many independent lines of strong evidence against the lactate shuttle model. Accordingly, lactate shuttling and utilization has remained a controversial issue (205–212). *In vitro* studies have shown that lactate can maintain ATP levels but not sustain neuronal signaling (213–217). These observations underscore the critical roles of glucose metabolism upstream of pyruvate/lactate for neuronal function.

It is well documented and uncontroversial that lactate may serve as a supplemental brain fuel when its blood level is elevated and exceeds the intracerebral level for example during exercise or lactate infusion (218–220). However, it is important to note that the metabolic efficacy of lactate supplementation depends on functional integrity of mitochondria (211). This fact has sometimes been overlooked which may lead to unfortunate clinical decisions (221, 222). A prior assessment of oxidative capability in each patient would be required if the metabolic benefits of lactate were to be evaluated.

Furthermore, it is important to remember that lactate transport across the BBB is an example of facilitated diffusion. The transport does not directly require chemical energy from ATP hydrolysis but the lactate molecules move down their concentration gradient. Accordingly, net transport of lactate into the brain requires that blood lactate level is higher than its intracerebral concentration. Thus, the recent report that transport of lactate into the brain may occur against a concentration gradient in brain trauma patients is erroneous (223). The observation is fully explained by the imprecision of the biochemical analytical biochemical techniques used (189).

As discussed previously, the BBB has a very low permeability for sodium (cf. Table 1). Accordingly, infusion of hypertonic sodium lactate is usually effective in reducing a dangerous increase in ICP (224, 225). If, in a patient with compromised CBF due to high ICP, hyperosmolar therapy decreases ICP, improvement of cerebral energy metabolism would be expected and does not indicate a specific beneficial metabolic effect of lactate infusion. For example, in a series of patients with cerebral hemorrhage and ICP > 20 mmHg, infusion of mannitol (1 g/kg) resulted in a significant decrease in ICP and cerebral LP ratio, increased CPP, and unchanged P_bt_O_2_ and concentration of cerebral glucose (226). In a situation of compromised cerebral energy metabolism and an increase of LP ratio, lactate flooding is associated with considerable risk. For example, it may inhibit glycolytic and pentose shunt fluxes in neurons and astrocytes and impair glycogenolysis in astrocytes (212).

### Bedside Evaluation of Cerebral Energy State—Future Possibilities

As the microdialysis probe reflects the biochemistry from a very narrow zone surrounding the dialysis membrane, appropriate positioning of the catheter in relation to focal lesions is necessary for a correct interpretation of the data obtained (193). During NCC, information regarding global cerebral energy state in addition to the regional information obtained from conventional intracerebral microdialysis would be valuable. Such information would also be of importance during critical care of other severe conditions when cerebral energy metabolism may be jeopardized without focal lesions (e.g., open-heart surgery, resuscitation after cardiac standstill, hemorrhagic or septic shock, toxic states). However, in these conditions, it is for various reasons difficult or impossible to insert intracerebral catheters. An alternative technique that avoids the penetration of cerebral tissue and still gives continuous bedside information regarding global cerebral energy state would be of apparent interest.

In a recent experimental study of induced hemorrhagic shock, the LP ratio in the draining cerebral vein (superior sagittal sinus) was compared to the LP ratio obtained from simultaneous intracerebral and intra-arterial microdialysis (227). In patients undergoing open heart surgery with cardio-pulmonary by-pass (CPB), a similar technique has been presented. The LP ratio obtained from microdialysis of the internal jugular vein increased significantly during CPB, indicating compromised cerebral oxidative metabolism, while conventional monitoring by NIRS did not show a corresponding decrease in cerebral oxygenation (228). Future studies will show whether the LP ratio obtained from draining cerebral venous blood will be informative enough to be used as a method to evaluate cerebral global energy state bedside.

Presently, the microdialysis technique used in clinical routine does not permit online monitoring. Today, the technique is laborious since it is necessary for the NCC personnel to transfer microvials at regular time intervals from the microdialysis catheter to the bedside analyzer. Techniques utilizing biosensors for true online monitoring are under development (229). By utilizing these techniques, it will probably in the near future be possible to monitor glucose, lactate, and pyruvate online bedside during NCC. If the LP ratio obtained from microdialysis of cerebral venous blood gives useful information regarding global cerebral redox state, we might in the near future have the possibility to monitor global cerebral energy state online. The technique might be used not exclusively during NCC but also during general intensive care where insertion of an intracerebral catheter is contraindicated or impossible.

### Summary: Cerebral Energy Metabolism

Since decades, the biochemistry of cerebral energy metabolism has been studied and documented in animal experiments. The microdialysis technique permits bedside monitoring of the most important biochemical variables during NCC. For a correct interpretation of the data, it is important to be aware of the prerequisites and limitations of the micordialysis technique and the biochemical analyses. The information obtained gives information regarding cerebral energy state and can be used to separate cerebral ischemia from mitochondrial dysfunction. However, the biochemical data obtained by intracerebral microdialysis are representative of a very small volume of tissue. Accordingly, a correlation to clinical outcome would be expected only when the volume studied is representative of a relatively large part of the brain. Future routine bedside monitoring of cerebral energy metabolism will probably depend on whether techniques permitting true online biochemical analysis by sensors are presented.

## Concluding Remarks

Neurocritical care is especially focused on problems related to ICP, CBF, and cerebral energy metabolism. Specific monitoring techniques have been introduced to give information regarding variables within these areas. To be of real importance for NCC, the techniques must give data that are obtained frequently/continuously, presented bedside and included in the clinical decision making. The information presented may be representative of a large part of the brain (global technique) or a very small volume surrounding the probe (focal technique). Both techniques have specific advantages and disadvantages. Many clinical studies have been devoted to examine the relation between the data obtained and clinical outcome. These efforts are not always motivated and may be questioned for several reasons. Most importantly, though physiological and biochemical data obtained by a global technique might be related to outcome, it is not justified to use data from a focal technique in this way unless it is known that these data are representative of a large part of the brain. Other clinical studies have examined possible correlations between the different physiological and biochemical techniques. Such correlations may be justified provided it is known that there is a physiological or biochemical relation between the variables. We will here briefly summarize our opinions regarding some of the techniques discussed previously.

### Global Physiological Techniques

Intracranial pressure is a keystone within NCC and CPP is calculated from ICP and the simultaneously monitored MAP. To be of clinical importance, ICP must be measured accurately and displayed continuously at the bedside. Presently, this may be achieved in two ways: from an intra-ventricular catheter or from a pressure-sensitive sensor placed in the brain.

The index named PRx is not related to a well-defined physiological mechanism or process, and the data obtained should not be regarded as a definite measure of vascular resistance or autoregulation. Several clinical studies have tried to correlate PRx with PbtO_2_ as well as with biochemical data obtained from microdialysis. As the mechanism behind PRx is not defined it is unclear why this global variable should be expected to correlate with any of these two focal techniques.

Under physiologically stable conditions, SjvO_2_ is interpreted as reflecting the balance between cerebral oxygen delivery and the cerebral metabolic rate of oxygen. However, the normal range of SjvO_2_ remains controversial, it provides limited information in patients with focal lesion, and technical monitoring problems are common. For obvious reasons, the correlation between SjvO_2_ and PbtO_2_ is often poor.

### Focal Physiological Technique

PbtO_2_ gives online information regarding tissue oxygen tension which is determined by the blood flow, the blood oxygen tension, and oxygen diffusion through the tissue. However, it does not disclose whether this oxygen tension is sufficient for maintaining adequate metabolism, and it has not been possible to define a threshold for PbtO_2_ below which hypoxic/ischemic cerebral damage occurs. As PbtO_2_ monitoring is a focal technique, a correlation to clinical outcome would be expected only when the data obtained reflects the situation in a relatively large part of the brain. The prerequisites for expecting a correlation between PbtO_2_ and LP ratio is discussed in the following paragraph.

### Focal Biochemical Technique

Presently, intracerebral biochemistry can only be evaluated bedside by utilizing microdialysis. During NCC routine, biochemical analyses can be used to give bedside information on cerebral energy status and signs of acute cellular degradation. In clinical praxis, it is important to use the technique in a standardized fashion which allows comparison between the data obtained and the levels observed in normal human brain. Routine biochemical analysis allows a clinically important separation of cerebral ischemia and mitochondrial dysfunction. The biochemical information obtained is representative of a very small tissue volume and a correlation to clinical outcome would be expected only when the conditions in this volume is representative of large part of the brain. As the LP ratio reflects cytoplasmatic redox state, a correlation to PbtO_2_ would be expected during cerebral ischemia but not during mitochondrial dysfunction.

### Global Biochemical Techniques

Presently, there is no technique available for evaluation of global cerebral energy state during NCC. Intraventricular insertion of the microdialysis catheter might be used in combination with conventional biochemical analysis, but the clinical benefits of this procedure have so far not been explored. Whether the LP ratio obtained from the draining cerebral venous blood might be informative is presently studied under experimental and clinical conditions. If the technique can be shown to give information regarding “global cerebral redox state,” it might be used to evaluate cerebral energy state also in clinical situations where insertion of an intracerebral catheter is risky or impossible.

### General Considerations for the Future

Future progress within NCC will necessitate a close collaboration between clinicians, experimental laboratories and companies developing new products for bedside monitoring and analysis. All physiological and biochemical interpretation should be based on solid data obtained in controlled experimental studies. It is important to realize that new biochemical principles will not be discovered under clinical conditions by utilizing routine bedside analytical techniques. For the intensivist working within NCC, it is already today a demanding task to interpret the interrelated and complex physiological and biochemical variables displayed bedside. In the future, this complexity will probably increase. To meet this development, the future intensivist will need deeper theoretical knowledge and, ideally, experiences from experimental studies within these specific areas of importance. Finally, the clinicians utilizing advanced monitoring techniques must understand the underlying principles and the limits and pitfalls associated with each specific method.

## Author Contributions

Conception and design of the review; drafting the work and revising the review critically for important intellectual content; final approval of the version to be published; agreement to be accountable for all aspects of the work ensuring that questions related to the accuracy or integrity of any part of the work are appropriately presented (C-HN, L-OK, and MO).

## Conflict of Interest Statement

The authors declare that the research was conducted in the absence of any commercial or financial relationships that could be construed as a potential conflict of interest.
